# 2025 Acknowledgment of *mSphere Ad Hoc* Reviewers

**DOI:** 10.1128/msphere.00850-25

**Published:** 2026-01-27

**Authors:** Ira Blader

**Affiliations:** 1 Biomedical Sciences and Pathobiology, Virginia-Maryland College of Veterinary Medicine 70732, Blacksburg, Virginia, USA

## EDITORIAL

As we approach the end of 2025, I have been reflecting on the past year’s events as well as how *mSphere* has moved forward. Scientific research, funding, and publishing have certainly seen more headlines in 2025 than any other year that I can remember. And while several headlines highlighted the best of what science can do, often we were greeted by unprecedented attacks on science and the scientific enterprise. Although I cannot speak for everyone, I think that it is safe to say that most of these stories have left us in shock and with trepidation over what science may look like in 2026 and beyond. Despite all this, microbiologists continue to show up. We are still working to make the world healthier and more sustainable by the research that we do as well as other activities that support and complement research, and this includes our reviewers. It is truly inspiring as an editor to see how many people readily accept our invitations to review a manuscript while the world surrounding us is hectic, chaotic, and stressful. Reviewers are the soul of any journal and are critical in helping us as a journal provide an outlet for scientists to publish their “news” or scientific discoveries. The fact that reviewers can do this while the world is metaphorically burning around them is a testament to their perseverance and dedication.

On behalf of the *mSphere* senior editors, Editorial Board, and staff, I want to thank the following individuals, who served as reviewers for the journal from December 2024 through November 2025:

Ghulam Abbas

Zack Abbott

Sabrina Absalon

Muhammad Afzal

Matthew Agler

Alain Bernardin Agnememel

Olusola Anuoluwapo Akanbi

Cesar G. Albarino

Emma Allen-Vercoe

Jontana Allkja

José M. Almendral

Francis Alonzo

Ingrun Alseth

Mohammed Alsharifi

J. Andrew Alspaugh

Craig Altier

Samuel Alvarez-Arguedas

John Alverdy

Stephanie J. Ambrose

Rocio Amorín De Hegedüs

Cheryl P. Andam

J. Christopher Anderson

Mark T. Anderson

Konstantin Andreev

Sergio O. Angel

Øystein Angen

Medini K. Annavajhala

Masaki Anraku

Haike Antelmann

Aileen Aoshoff

Amir Arastehfar

Linda S. Archambault

Benjamin Aroeti

Fiorentina Ascenzioni

Luís Augusto Nero

Michael A. Bachman

Maria Bagattini

Sue Baigent

Frederik Bak

Erin Baker

Gabriela Balikova Novotna

Hannah J. Barbian

Daniel Barkan

Francisco Barona Gómez

Christine Marie Bassis

Joyoti Basu

Dwayne M. Baxa

Arden Baylink

Özgür Bayram

Inga Bazukyan

Daan Beentjes

Samantha L. Bell

Mark Benedict

Maria Soledad Benitez

Jane Claryn Benjamin

Teresa M. Bergholz

Hanna Leah Berman

Ananda Bhattacharjee

Disha Bhattacharjee

R. Blake Billmyre

Marco Binder

Svend Birkelund

Susan E. Birket

Karishma Bisht

Indranil Biswas

Matthew G. Blango

Elisa Blinda

Pavel Bobrovsky

Lydia M. Bogomolnaya

Francois-Michel Boisvert

Mariangela Bonnizoni

Eric M. Bottos

Dawn M. E. Bowdish

Prosper Boyaka

Angela K. Boysen

Patricia A. Bradford

Volker Briken

Françoise Bringel

Andrew James Broadbent

Kenneth Brockman

Christopher B. Brooke

Heike Brötz-Oesterhelt

Ian H. Brown

Patrick Jeffrey Browne

John H. Brumell

Mary Ann V. Bruns

Bryan D. Bryson

Dengpan Bu

Lindsey Burcham

Silvia Buroni

Laura S. Burrack

Ruben D. Cadena-Nava

Laty Adriella Cahoon

Yi Cai

Kenneth Campellone

Maria Jorge Campos

Kyle J. Card

Andrés Carrazco Montalvo

Ronan K. Carroll

Monica Cartelle Gestal

Arturo Casadevall

Santiago Castillo-Ramírez

Meaghan Castledine

Clayton C. Caswell

Jean Celli

Kateřina Černá

Benjamin Chadwick

Serafeim C. Chaintoutis

Ankur Chakravarthy

Josephine R. Chandler

Jeff H. Chang

Theresa L. Chang

Dipankar Chatterji

Alfredo Chavez-Arroyo

Changbin Chen

Chen Chen

Chia-Yu Chen

Ji-Ming Chen

Juanjuan Chen

Ko-Hsuan Chen

Liang Chen

Qing Chen

Qu Chen

Loo Wee Chia

Chuan Chiang-Ni

Delma Childers

You-Hee Cho

Oana Ciofu

Christine Citti

Sarah E. Clark

Carolina Coelho

Maria Carmen Collado

Sarah S. Comstock

Sean Conlan

Brian Conlon

Brendan Cormack

Deiziane Da Silva Costa

Wellington Felipe Costa

Margaret K. Costello

Matthew L. Cotten

Pauline M. L. Coulon

Loic Coutte

Robert Cox

Hannah M. Creager

Peter Cronin

Jie Cui

Paul J. Cullen

Eddie Cytryn

Jianfeng Dai

Denis Marc Daigle

F. Heath Damron

Antoine Danchin

Pranav Danthi

Viet Loan Dao Thi

Anindya Das

Saumitra Das

Bryan W. Davies

Faith A. Davis

Andrew Day

Kristen M. Deangelis

Eliana De Gregorio

Henk C. Den Bakker

Zhaochao Deng

Nathaniel David Denkers

Bruno Francesco Rodrigues De Oliveira

Terence S. Dermody

Perumal Arumugam Desingu

Julie Deslippe

Dewa A. P. Rasmika Dewi

Manpreet K. Dhami

Sayera Dhaubhadel

Sourabh Dhingra

Samuel L. Díaz-Muñoz

Laura Dickson

Kimberly Anne Dill-Mcfarland

Nicholas Dimonaco

Marc S. Dionne

Richard D. Dix

Eunsoo Do

Wiliam T. Doerller

Yohei Doi

Pavel Dolezal

Nazzareno Dominelli

Scott Jorge Dos Santos

Shuai Du

Wenqi Du

Lidiya Dubytska

Edward G. Dudley

Breck A. Duerkop

Nisha K. Duggal

Ildiko R. Dunay

David Duneau

Nelita Du Plessis

Jeffrey D. Dvorin

Hanareia Ehau-Taumaunu

Mikael Elias

Waldir P. Elias

Marie A. Elliot

Iuliana V. Ene

Jan Engelstaedter

Steven Estus

Sarah Elisabeth Ewald

Camilla Fagorzi

Chia-Kwung Fan

Hongjie Fan

Yaxin Fan

Michael J. Federle

Lorena Fernandez-Martinez

Kenneth A. Fields

Daniel H. Fine

Reinhard Fischer

Skye R. S. Fishbein

Derek J. Fisher

Ross Fitzgerald

Anthony R. Flores

Ana L. Flores-Mireles

Matthew H. Foley

Steven L. Foley

Jarrod R. Fortwendel

Derek Foster

Timothy J. Foster

Dawn Foster-Hartnett

Lydia Freddolino

Jeffrey A. Freiberg

Nancy E. Freitag

Matthew B. Frieman

Ernesto J. Fuentes

Naoki Fukuma

Kevin Fuller

Michaela Ulrike Gack

Jennifer Gaddy

Ketaki Ganti

Michael G. Ganzle

Fangzhou Gao

Hanxing Gao

Erin C. Garcia

Miguel García-Knight

Amit Gaurav

Uma Shankar Gautam

Michael J. Gebhardt

Angie Gelli

Kathleen Gensheimer

Mathieu Gissot

David Goldblatt

James W. Golden

Erin D. Goley

Jun Gong

Xianzhe Gong

Yu-Nong Gong

Lisandro J. González

Andrew Gorringe

Joerg Graf

Lone Gram

Elizabeth A. Grice

Marc-Jan Gubbels

Lianxian Guo

Xue Guo

Francisco Gutierrez-Santiago

Max M. Haggblom

Andrea Hahn

Sandra K. Halonen

Michael R. Hamblin

Trinity L. Hamilton

Neal D. Hammer

Kyudong Han

Shrikant Harne

Joe Harrison

Karl A. Hassan

Masashi Hatamoto

Asma Hatoum-Aslan

Kuang He Kuang

Adriano O. Henriques

Melissa M. Herbst-Kralovetz

Sander Herfst

Syrie Hermans

Hoa Thi Thanh Hoang

Linda M. Holland

Jeffrey Holloman

Geoffrey H. Holm

Christoph Hölscher

Galadriel Astra Hovel-Miner

Daniel K. Howe

Fupin Hu

Pengjie Hu

Da Huang

Guanghua Huang

Kaisong Huang

Manning Huang

Karthik Hullahalli

Michael J. Imperiale

Yoshikazu Ishii

Denis Jallet

Kehrmann Jan

Manon Janet-Maitre

Victoria Jeffers

Ann E. Jerse

Jian-Yu Jiao

Zhang Junya

Manabu Kanno

Moira Kelly

Robyn S. Kent

Lee J. Kerkhof

Jemila Caplan Kester

Fazlurrahman Khan

Megan R. Kiedrowski

Hwan Keun Kim

Jae-Ouk Kim

Min-Ju Kim

Dustin King

Laura J. Knoll

Gregory D. Koblentz

Shilpa Kodati

Tamar Kohn

James B. Konopka

Jennifer Houle Kopanke

Daniel Kornitzer

Anita A. Koshy

Joanna Koziel

Petya Violinova Krasteva

Christopher J. Kristich

James W. Kronstad

Damian J. Krysan

Kengo Kubota

Ranjit Kumar

Sanjeev Kumar

Eric R. Lafontaine

Kristen Landreville

Andrew S. Lang

Christopher N. Larock

Martin Frederik Laursen

Vincent T. Lee

Elliot J. Lefkowitz

Ruipeng Lei

Danielle G. Lemay

Nicholas J. Lennemann

Karine G. Le Roch

Valerie Le Sage

Pok Man Leung

Gene-Wei Li

Mingkun Li

Na Li

Ruichao Li

Shuzhen Li

Tao Li

Yeqi Li

Zhu-Hong Li

Chunxiao Li

Guodong Liang

Julian Liber

Sabine Lichtenegger

Nicole A. P. Lieberman

Shen Jean Lim

Ching-Hsuan Lin

Xiangtian Ling

Haoping Liu

Keshao Liu

Monica Liu

Sen Liu

Shuangping Liu

Nicolas Locker

Melissa Bruckner Lodoen

Png Loke

Matthew E. Long

Zexu Long

Xavier Lopez-Labrador

Michael C. Lorenz

Chunzhe Lu

Ling Lu

Ti Lu

Yang Lu

Arthur J. Lustig

Jonathan B. Lynch

Liping Ma

Xiang Ma

Jessie MacAlpine

Milton Maciel

Matthias Mack

Suresh Mahalingam

Lucie Anne Malard

Harmit S. Malik

Mark J. Mandel

Nicholas J. Mantis

Chinmay Mantri

William Margolin

José Maria Marimón

Jon Marles-Wright

Laetitia Maroc

Ian Marriott

A. James Mason

Joseph J. Mattapallil

Joshua T. Mattila

Tim McAllister

John K. McCormick

John T. McCrone

Patrick McGann

Rachel M. McLoughlin

Nigel McMillan

David N. McMurray

Vineet D. Menachery

Julie L. Meyer

Antonella Migliaccio

Aaron W. Miller

William E. Miller

Kingston H. Mills

Richa Mishra

Aaron P. Mitchell

Michelle Momany

Silvia Monteiro

Kyung Moon

Renata Moreno

Yasuhiro Morita

James C. Morris

Joachim Morschhäuser

Daisuke Motooka

Katharina Mueller

Volker Mueller

Katsuhiko Murakami

Eain A. Murphy

Theodore Muth

Nontobeko Eunice Mvubu

Cullen Lucan Myers

Saisubramanian Nagarajan

Moon H. Nahm

Ichiro Nakagawa

Paola Navarrete

Fernando Navarro-Garcia

Boris Negrutskii

Nhu Nguyen

Kuikui Ni

Marisa C. Nielsen

Masahiro Niikura

Tomoki Nishioka

Ben Niu

Peter Nixon

Justin R. Nodwell

Steven J. Norris

Eric L. Nuermberger

Charles Nyagupe

Javier Ochoa-Repåraz

Amanda G. Oglesby

John Okombo

Cheryl Y. M. Okumura

Antonio Oliver

Eoghan O'Neill

Eng Eong Ooi

Mona Orr

Karen M. Ottemann

Scot P. Ouellette

Joerg Overhage

Michelle A. Ozbun

Aseem Palande

Guy H. Palmer

Kelli L. Palmer

Costas C. Papagiannitsis

Bhavya Papudeshi

Daniel Paredes-Sabja

Ivana Parker

Colin R. Parrish

Sally R. Partridge

Kathryn A. Patras

Dimitri Pestov

Brian Peters

Brian M. Peters

Tracey Lee Peters

Lauren Petrullo

Eugen Pfeifer

Tuyetnhu Pham

Isabelle Q. Phan

Francesca Pittino

Spyros Pournaras

Cristina Povedano-Priego

Arthur Prindle

Sladjana Prisic

Firdausi Qadri

Ping Qian

Shangshang Qin

Janet Quinn

Camilo Quiroga-González

Alaa Emara Rabee

Lilliana Radoshevich

Sima Rafati

Kathryn C. Rahlwes

Govindan Rajamohan

Jasna Rakonjac

Francisco Ramos-Morales

Jolene Ramsey

Tara M. Randis

Yang-Zhi Rao

Viduthalai Rasheedkhan Regina

David Rasko

Kate Reddington

E. Hesper Rego

Andy Reisinger

Haiwei Ren

Jose M. Requena

Oleg N. Reva

Gabriela Fior Ribeiro

Ezio Ricca

Jonathan P. Richardson

Douglas Risser

Fabian Rivera-Chávez

Marina Campos Rocha

Andjela Rodic

Eduardo Rodríguez-Román

P. David Rogers

Kyle H. Rohde

Amy Rohlfing

Alicia Rojas

Louise A. Rollins-Smith

James C. Romero-Masters

Severin Ronneau

R. Martin Roop

Cally Roper

Bruce A. Rosa

Sarah Elizabeth Rowe

Hang Ruan

Zhi Ruan

Adam D. Rudner

Alessia Ruggieri

Daniel Ruzek

Jeffrey M. Rybak

Kuttalingam Sabarinathan

Ranjodh Sandhu

Carlos Sandoval-Jaime

Tasha Santiago-Rodriguez

Kaustuv Sanyal

Noreen Sarwar

Akira Sasaki

Yoshiaki Sato

Kamaraj Sattu

Karin Sauer

Karen M. Scanlon

Lennart Schada Von Borzyskowski

Michael Schindler

Thomas M. Schmidt

David A. Schneider

Tony Schountz

Oliver Schwengers

Anna Maria Seekatz

William M. Shafer

Shuai Shao

Yongqi Shao

Lindsey N. Shaw

Michael J. Sheedlo

Aimee Shen

Bang Shen

Zunji Shi

Erika Shor

Charlotte-Eve Short

Francesca L. Short

Bupe A. Siame

Ella T. Sieradzki

Nathan Simon

Baneshwar Singh

Sukrit Singh

Ritam Sinha

Jerod A. Skyberg

James M. Slauch

Ray T. Y. So

Min-Suk Song

Rodolpho Souza

Andrea Spitaleri

Laura St Clair

Madison E. Stellfox

Sandra Suescun

Akifumi Sugiyama

Keer Sun

Elena Suvorova

Haruo Suzuki

Shunya Suzuki

Marc Swidergall

W. Edward Swords

Kapil Tahlan

Norio Takeshita

Aimee Tan

Demeng Tan

Shumin Tan

Qi Wen Teo

Anil Thakur

Shankar Thangamani

Vinai Chittezham Thomas

Shailly Tomar

Chris J. Tonkin

Shotaro Torii

Cristina Torres Fuentes

Arthur H. Totten

Dieter M. Tourlousse

Greg J. Towers

Ana Traven

Krzysztof Trzciński

Argyro Tsipa

M. F. Tuite

Michael J. Turell

Kevin M. Tyler

Gregory Tyrrell

Taher Uddin

Miguel A. Valvano

Koenraad Van Doorslaer

Douwe van Sinderen

Daria Van Tyne

Christopher N. Vassallo

Arundhathi Venkatasubramaniam

Guilherme G. Verocai

Jay Vornhagen

Daniel E. Voth

Bao Gia Vu

William G. Wade

Kimberly A. Walker

Guangshun Wang

Hualong Wang

Jie Wang

Junjian Wang

Luoyang Wang

Yonglong Wang

Yuguang Wang

Gary E. Ward

Matthew J. Wargo

Omar Warsi

Erica J. Washington

Jeremy S. Webb

Guangshan Wei

Tiffany Weinkopff

Allison Weis

Daniela Weiskopf

Brian James Werth

Michael R. Wessels

Christopher M. West

Alexander Westermann

Katherine Wetzel

Robert T. Wheeler

Travis Wiles

Christiane E. Wobus

Nicholas A. Wood

Michael H. Woodworth

Yanxia Wu

Nelson Arno Wulff

Bin-Bin Xie

Jianping Xie

Xianjie Xiu

Masahiro Yamashita

Bo Yan

Xianqin Yang

Xinyi Yang

Zhaomin Yang

M.-N. Frances Yap

Phillip Yates

Akihiro Yoshida

Xuefei Yu

Yunsong Yu

Andrew D. Yurochko

Jose Yuste

Geoffrey Zahn

Michael A. Zeller

Anna C. Zemke

Lanying Zeng

Yuan Zeng

Fushun Zhang

Jun Zhang

Kai Zhang

Keke Zhang

Lu Zhang

Shizhu Zhang

Xue Zhang

Yuanwei Zhang

Zhenjie Zhang

Chunyi Zhou

Annelies S. Zinkernagel

Soumaya Zlitni

Sadri Znaidi

Ibrahim Zuniga-Chaves

